# A High-Sensitivity Gravimetric Biosensor Based on S_1_ Mode Lamb Wave Resonator

**DOI:** 10.3390/s22155912

**Published:** 2022-08-08

**Authors:** Tiancheng Luo, Wenjuan Liu, Zhiwei Wen, Ying Xie, Xin Tong, Yao Cai, Yan Liu, Chengliang Sun

**Affiliations:** 1 The Institute of Technological Sciences, Wuhan University, Wuhan 430072, China; 2 Hubei Yangtze Memory Laboratories, Wuhan 430205, China

**Keywords:** biosensor, high-sensitivity, Lamb wave, S_1_ mode

## Abstract

The development of MEMS acoustic resonators meets the increasing demand for in situ detection with a higher performance and smaller size. In this paper, a lithium niobate film-based S_1_ mode Lamb wave resonator (HF-LWR) for high-sensitivity gravimetric biosensing is proposed. The fabricated resonators, based on a 400-nm X-cut lithium niobate film, showed a resonance frequency over 8 GHz. Moreover, a PMMA layer was used as the mass-sensing layer, to study the performance of the biosensors based on HF-LWRs. Through optimizing the thickness of the lithium niobate film and the electrode configuration, the mass sensitivity of the biosensor could reach up to 74,000 Hz/(ng/cm^2^), and the maximum value of figure of merit (FOM) was 5.52 × 10^7^, which shows great potential for pushing the performance boundaries of gravimetric-sensitive acoustic biosensors.

## 1. Introduction

The in situ detection of biomolecules plays a very important role in applications such as disease diagnosis, environmental monitoring, and food security analysis [1]. This demand has become more apparent and urgent after the outbreak of the novel coronavirus (COVID-19). Among the various detection techniques [2,3], MEMS acoustic biosensors have become a research hotspot because of their small size, real-time response, and high sensitivity, which are the key drivers of wide-spread usage.

MEMS technology satisfies the key requirement of biosensors of miniaturization while keeping a high sensitivity [4]. Quartz crystal microbalance (QCM) [5,6,7], surface acoustic wave (SAW) resonators [8,9,10], and film bulk acoustic wave resonators (FBAR) [11,12,13,14] are the three main types of acoustic biosensors that have been extensively studied [15,16]. Although their sensitivity is not very high, QCM as a gravimetric-sensitive biosensor is popular because of their simplicity and low-cost characteristics. SAW is the most sensitive to surface changes as an acoustic sensor in gas sensing. However, strong acoustic radiation limits the application of SAW in liquid environments, so shear horizontal SAW and love SAW were developed to reduce the acoustic radiation. Shear mode FBAR [17] works at a much higher frequency, due to its very thin piezoelectric stack thickness, which determines the resonance frequency. Despite its high frequency and high sensitivity, the application of FBAR is much costlier, because the fabrication process is complex.

Recently, a potential method was proposed to suppress acoustic radiation and improve the mass sensitivity in water at high frequencies. This idea was exploited in biosensors based on Lamb wave resonators (LWRs) [18,19,20,21]. Similarly to other acoustic wave resonators, the application of Lamb wave resonators in mass sensing mainly depends on the frequency shift of the device. The frequency of LWRs [22,23] can be defined by, not only the thickness of the piezoelectric film, but also the configuration of their interdigital electrodes (IDTs) [24,25]. When surface acoustic waves are guided laterally into a sufficiently thin plate, they are referred to as Rayleigh–Lamb waves or Lamb waves. Generally, the different plate modes are denoted as S_n_ or A_n_, representing the nth order symmetric or asymmetric Lamb wave modes, where “n” is an integer ranging from 0 to infinity and represents the number of standing waves along the plate thickness [26].

Thanks to film transfer technology [27,28], LiNbO_3_ or LiTaO_3_ thin film-based acoustic resonators have been widely studied [29,30]. The S_1_ mode LWR is strong at vertical direction and can enable a high resonance frequency and large coupling coefficient simultaneously [31]. Recently, S_1_ mode resonators with ultra-high quality factor (*Q*) based on lithium niobate (LN) film have been fabricated [32]. These findings demonstrate the great advantages of S_1_ Lamb wave resonators for biosensing applications. Their high-frequency characteristics make the devices immune to low-frequency magnetic noise. For biosensors working in a liquid environment, the energy loss is very large, and the measurement range of a biosensor with a low *Q* value will become smaller or the device will even fail immediately.

In this paper, S_1_ mode high frequency Lamb wave resonators (HF-LWR) based on X-cut LN film were simulated and fabricated. We investigated the influences of the thickness of the LN film and the IDTs configuration on the performance of the resonators. Moreover, the feasibility of HF-LWRs as biosensors in an liquid environment was studied. The results showed that a biosensor based on HF-LWR has an ultra-high mass sensitivity and figure of merit (FOM).

## 2. Resonators

The performance of piezoelectric resonator-based sensors strongly depends on the design of the resonator. The operating frequency, sensitivity, resolution, etc. of the sensor are closely related to the performance of the resonator. In this section, an S_1_ mode resonator in X-cut LiNbO_3_ thin film is studied using a combination of theoretical design, finite element analysis, and experimental verification.

**Figure 1:**
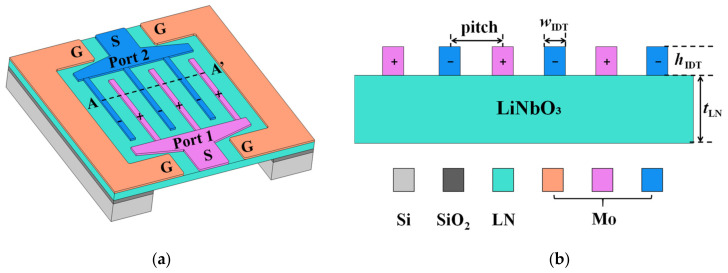
(**a**) The structural design of HF-LWR based on LN film; (**b**) Cross-sectional view of the active area of the resonator and the parameters studied.

Figure 1a shows the structural design of the HF-LWR. The device comprises a LN thin film and two IDT arrays with different voltage polarities. Port ‘S’ is connected to signal and port ‘G’ is connected to ground. Molybdenum (Mo) was chosen as the material for the electrode, to minimize the acoustic attenuation and to provide good electrical conductivity [33,34]. The silicon dioxide under the LN film was used as a bonding layer in the fabrication process. Figure 1b shows a cross-sectional view of the active area (along dotted line AA’ in Figure 1a) of the resonator and parameter settings. Pitch is the IDTs periodic length. $t_{\mathrm{LN}}$ is the thickness of LN thin film, while $w_{\mathrm{IDT}}$ and $h_{\mathrm{IDT}}$ are the width and height of the IDTs, respectively.

**Figure 2:**
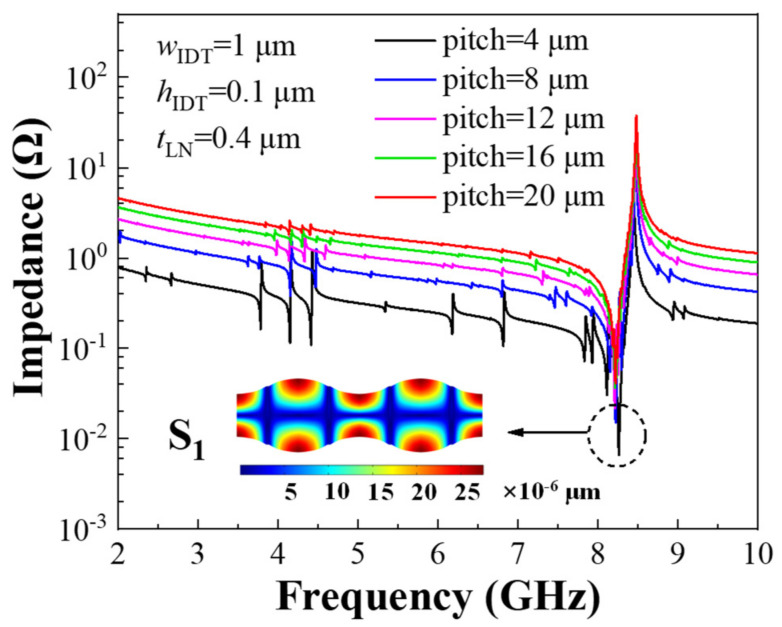
Impedance curves of resonators with different IDT pitches; the inset is the resonator displacement at resonance frequency.

Figure 2 shows the impedance curves of resonators with different IDT pitches. Due to the high phase velocity of the Lamb wave S_1_ mode, its operating frequency can be higher than 8 GHz on the LN film. Resonators with different pitches all excite the S_1_ mode. Since the S_1_ mode is vertical propagation, optimization of the pitch changes both the resonance frequency (*f*_s_) and anti-resonance frequency (*f*_p_) little. Spurious modes in resonators need to be suppressed as much as possible, to ensure data accuracy in mass sensing applications. As can be seen from the impedance curve in Figure 2, the design with a large pitch has fewer spurious modes. In addition, the curve at *f*_p_ is smoother than at *f*_s_, which means that *f*_p_ is more suitable as a reference standard for the biosensor frequency shift.

**Figure 3:**
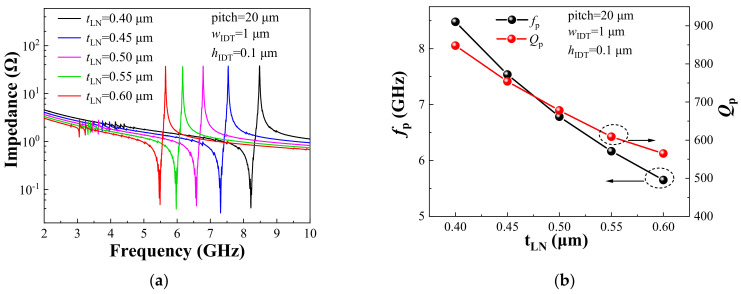
(**a**) Impedance curves of HF-LWRs with different $t_{\mathrm{LN}}$; (**b**) Influences of $t_{\mathrm{LN}}$ on *f*_p_ and *Q*_p_.

Figure 3a shows the impedance curves of HF-LWRs with different $t_{\mathrm{LN}}$. Figure 3b shows the influences of $t_{\mathrm{LN}}$ on *f*_p_ and *Q*_p_. The *f*_p_ of the resonator is inversely proportional to the $t_{\mathrm{LN}}$. Hence, the design of the thin film can shield some low-frequency magnetic noise. In addition, the *Q*_p_ of the resonator is also inversely proportional to the $t_{\mathrm{LN}}$. For resonators, higher *Q* values represent lower energy losses. Although the thinner the piezoelectric film, the greater the *Q* value, there are still some other constraints that need to be considered in a design. A device with a very thin film becomes very fragile, especially when operating in a liquid environment. Moreover, a too thin film will deteriorate the performance of the piezoelectric film and increase the difficulty of the fabrication process.

**Figure 4:**
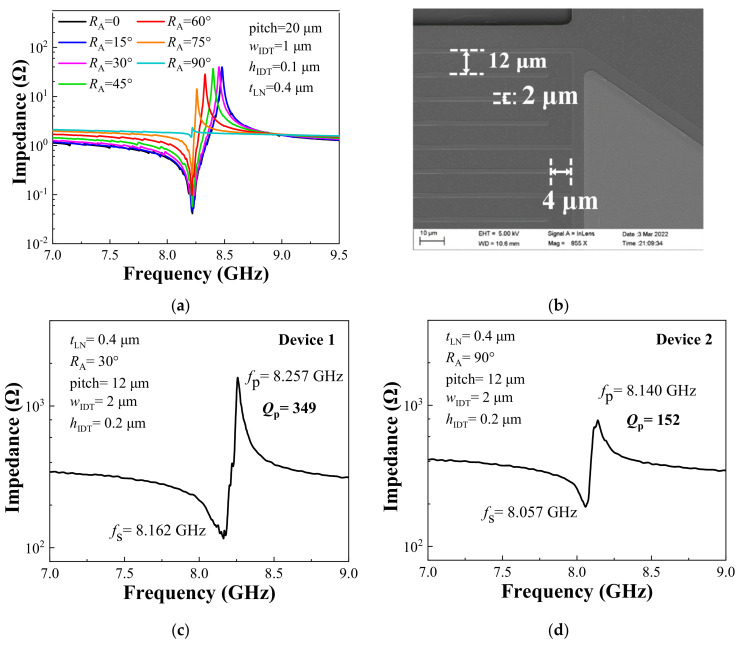
(**a**) Impedance curves of resonator simulation with different IDTs rotation angles; (**b**) SEM image of the fabricated resonators; Measured results of (**c**) device 1 and (**d**) device 2.

Figure 4a shows the influences of IDTs rotation angle (*R*_A_) on the performance of the resonator. The optimization of the IDTs rotation angle has a large impact on the *f*_p_, while the *f*_s_ is almost constant. Figure 4b is an SEM image of the fabricated resonators. Considering both the design requirements and the feasibility of the process, the HF-LWRs were fabricated in 400-nm-thick X-cut LiNbO_3_ thin film, and the height of the IDTs was 200 nm. We designed a strip-shaped groove at both ends of the active region, to enhance the *Q* value of the device. Figure 4c,d shows the impedance curves of two measured resonators with different *R*_A_. Due to certain deviations in the film thickness of the entire wafer, there was a slight deviation in the frequency of different devices. However, for the operating frequency over 8 GHz, the frequency deviation of about 1.3% was small enough to be ignored. In general, the experimental data demonstrated the accuracy of the design of the HF-LWR. Moreover, the testing results showed that the *Q*_p_ of the resonator was small when the IDTs rotation angle was 90°, which needs to be avoided in the design of a sensor.

## 3. Biosensors

**Figure 5:**
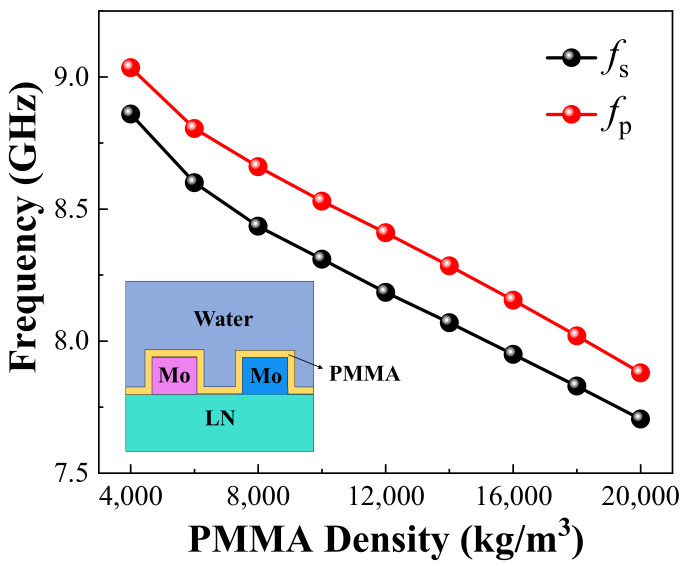
Variation of *f*_s_ and *f*_p_ with different PMMA densities. The inset is the structural design of biosensor based on HF-LWR.

In this section, we study the performance of the biosensors based on HF-LWRs using a finite element analysis. A 50-nm polymer layer (Polymethyl methacrylate, PMMA) was used as the mass sensing layer [18,35]. It uniformly covered the surface of the HF-LWR, as shown in the inserted image of Figure 5. In order to simulate the gravimetric change that occurs when a sensor adsorbs biomolecules in water, we studied the effect of the density change of PMMA on the frequency shift of the device. From Figure 5 we can see that the *f*_s_ and *f*_p_ of the sensor decreased synchronously as the PMMA density increased.

To evaluate the mass sensitivity of the device, we changed the density of the sensing layer and recorded the corresponding frequency shift. The variation of the density is an emulation of the gravimetric loading effect when the biosensor absorbs biomolecules. The mass sensitivity *S*_m_ is calculated with the following equation:

(1) $$S_{m}=\left|\frac{\Delta f}{T_{s}\Delta \rho_{s}}\right|$$

where Δf is the frequency shift with different gravimetric loadings, and $T_{s}$ and $\Delta \rho_{s}$ are the thickness and density variation of the sensing layer, respectively.

**Figure 6:**
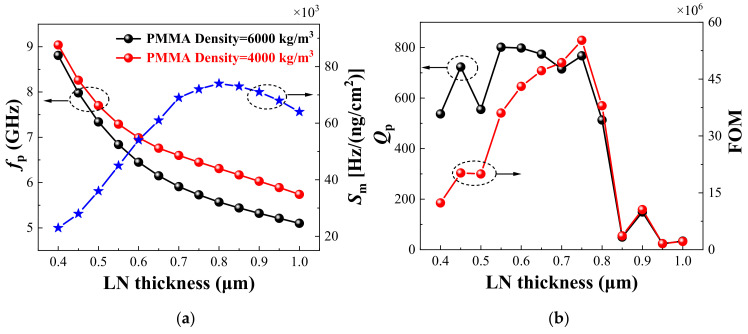
Influence of LN film thickness on (**a**) *f*_p_, *S*_m_, and (**b**) *Q*_p_, FOM of biosensors.

Figure 6a shows the variation of *f*_p_ and *S*_m_ with different LN film thicknesses. Similarly, the red and black curves represent the variation of the *f*_p_ with the LN film thickness when the PMMA density was 4000 kg/m^3^ and 6000 kg/m^3^, respectively. From 0.4 μm to 1 μm, with the increase of LN film thickness, the *f*_p_ of the device gradually decreased, while the decrease rate was changing, which affected the *S*_m_. The blue curve represents the variation of the *S*_m_ with different LN film thicknesses. From the figure we can see that *S*_m_ reached the maximum value of 74,000 [Hz/(ng/cm^2^)] when the LN film thicknesses was 0.8 μm.

Besides sensitivity, *Q* value and FOM are also very important parameters for evaluating the performance of a sensor. *Q* depends on the energy loss of the biosensor in water, and the FOM is defined as the mass sensitivity multiplied by the quality factor, which directly indicates the performance of the device [36]. The *Q* value and FOM of the biosensor can be calculated using the following equation:

(2) $$Q=\frac{f}{2}\frac{d∠Z}{df}\;$$

(3) $$\mathrm{FOM}=S_{m}\times Q$$

where Z is the electrical impedance of the device.

Figure 6b shows the influence of LN film thickness on the *Q*_p_ and FOM of the biosensors. When the thickness of the LN film was 0.55 μm, the *Q*_p_ of the biosensor achieved the maximum value of 801. When the LN film thickness was greater than 0.75 μm, there was a steep drop in the *Q*_p_ of the biosensor, due to the increased loss. The changing law of the FOM value depends on the superposition of *S*_m_ and *Q*_p_. When the thickness of the LN film was 0.75 μm, the FOM of the biosensor achieved the maximum value of 5.52 × 10^7^. Compared with the sensitivity and *Q* value, the FOM directly reflects the overall performance of the sensor, which provides guidance for the design of the sensor.

**Figure 7:**
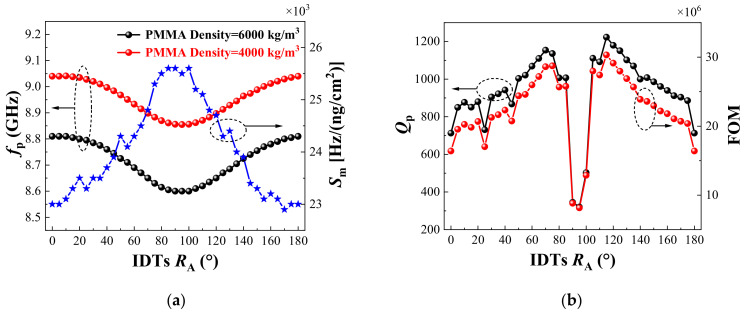
Variation of (**a**) *f*_p_, *S*_m_, and (**b**) *Q*_p_, FOM of biosensors with different IDTs rotation angles.

As shown in the measured result in Figure 4, the IDTs rotation angle strongly affected the device. Figure 7a shows the variation of *f*_p_ and *S*_m_ with different IDTs rotation angles. The red and black curves represent the variation of *f*_p_ with the IDTs rotation angle, when the PMMA density was 4000 kg/m^3^ and 6000 kg/m^3^, respectively. With the change of the IDTs rotation angle, the two curves had the same change law, and the difference between them represented the sensitivity of the biosensor. In the range of 0 to 90 degrees, the frequency of the biosensor decreased gradually as the IDTs rotation angle increased. However, due to the symmetry of the rotation angle of IDTs, there was an opposite trend in the range of 90 to 180 degrees. The blue curve represents the variation of the *S*_m_ with different IDTs rotation angles. Contrary to the changing trend of *f*_p_, *S*_m_ reached the maximum value 25,600 (Hz/(ng/cm^2^)) when the IDTs rotation angle was 90 degrees.

The black curve in Figure 7b shows the influence of the IDTs rotation angle on the *Q*_p_ of the biosensor. When the IDTs rotation angle was 115 degrees, the *Q*_p_ of the biosensor achieved the maximum value of 1223. When the IDTs rotation angle was from 0 to 70 degrees, the *Q*_p_ of the device basically showed an increasing trend. However, when the rotation angle was around 90 degrees, the *Q*_p_ of the device had an obvious, sharp drop. While the rotation angle was in the range of 90 to 180 degrees, *Q*_p_ presented a symmetrical change. The red curve in Figure 7b shows the influence of the IDTs rotation angle on the FOM of the biosensor. As the change of *S*_m_ with the IDTs rotation angle was small, the change of FOM value was basically consistent with the change of *Q*_p_. When the thickness of the IDTs rotation angle was 115 degrees, the FOM of the biosensor achieved the maximum value of 3.03 × 10^7^.

## 4. Conclusions

In this paper, we simulated and fabricated HF-LWRs based on X-cut LN film. The resonator comprised a LN thin film and two IDT arrays on the same side of the piezoelectric film, and the S_1_ mode of Lamb wave with a high phase velocity was excited. The influences of different thicknesses of LN film and IDT configurations on the performance of the devices were investigated using a theoretical analysis and finite element analysis simulation. Using thin film transfer technology, we fabricated a batch of resonators, and the measured resonance frequencies were all above 8 GHz. Although there was a small deviation (about 1.3%) in the frequency of the device, due to the thickness uniformity of wafer, the experimental data of the fabricated devices also showed a good agreement with the simulation results. Furthermore, we studied the performance of the biosensors based on HF-LWRs using a finite element analysis. A 50-nm-thick PMMA was used as the mass sensing layer. The mass sensitivity of the biosensors based on HF-LWRs could reach a high level, due to the high frequency characteristics of the resonators. By optimizing the parameters of the biosensors, the maximum mass sensitivity reached 74,000 (Hz/(ng/cm^2^)), and the maximum FOM value reached 5.52 × 10^7^. This work on acoustic resonators and biosensors has great potential for high-sensitivity gravimetric biosensing in applications such as disease diagnosis, environmental monitoring, and food security analysis.

## Data Availability

Not applicable.
